# *catena*-Poly[[[bis­(methanol-κ*O*)bis­(seleno­cyanato-κ*N*)manganese(II)]-μ-1,2-bis­(pyridin-4-yl)ethane-κ^2^ *N*:*N*′] 1,2-bis­(pyridin-4-yl)ethane monosolvate]

**DOI:** 10.1107/S1600536813007150

**Published:** 2013-03-20

**Authors:** Susanne Wöhlert, Inke Jess, Christian Näther

**Affiliations:** a Institut für Anorganische Chemie, Christian-Albrechts-Universität Kiel, Max-Eyth-Strasse 2, 24118 Kiel, Germany

## Abstract

The reaction of manganese seleno­cyanate with 1,2-bis­(pyridin-4-yl)ethane (bpa) leads to the title compound, {[Mn(NCSe)_2_(C_12_H_12_N_2_)(CH_3_OH)_2_]·C_12_H_12_N_2_}_*n*_. The Mn^II^ cation is coordinated by two *N*-bonded seleno­cyanate anions, two bpa ligands and two *O*-bonded methanol mol­ecules, within a slightly distorted octa­hedral geometry. The Mn^II^ cations and the non-coordinating *N*-donor ligands are located on centers of inversion while the coordinating *N*-donor co-ligands are located on a twofold rotation axis. In the crystal, the Mn^II^ cations are linked into chains along the *c*-axis direction by the bpa ligands. The chains are further connected *via* a non-coordinating bpa ligand into layers parallel to (3-10) *via* O—H⋯N hydrogen-bonding inter­actions.

## Related literature

For background to this work and the structures of related compounds, see: Boeckmann & Näther (2010 ▶, 2012 ▶), Wöhlert *et al.* (2012 ▶), Wöhlert & Näther (2012*a* ▶,*b* ▶).
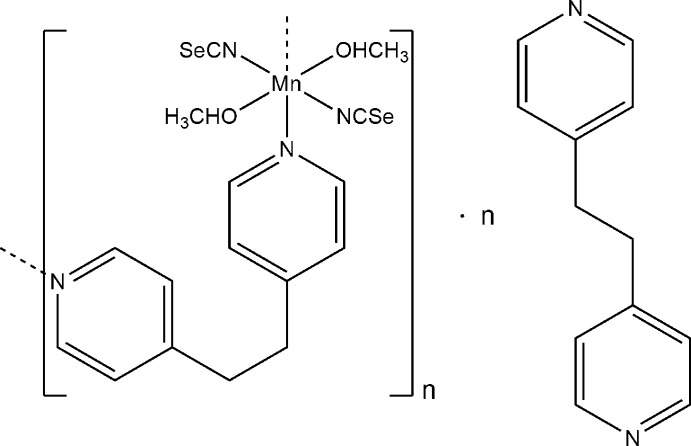


## Experimental

### 

#### Crystal data

[Mn(NCSe)_2_(C_12_H_12_N_2_)(CH_4_O)_2_]·C_12_H_12_N_2_
*M* *_r_* = 697.46
Monoclinic, 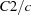

*a* = 19.184 (1) Å
*b* = 9.7854 (4) Å
*c* = 17.3468 (9) Å
β = 108.624 (4)°
*V* = 3085.9 (3) Å^3^
*Z* = 4
Mo *K*α radiation
μ = 2.82 mm^−1^
*T* = 293 K
0.14 × 0.11 × 0.06 mm

#### Data collection

Stoe IPDS-2 diffractometer
Absorption correction: numerical (*X-SHAPE* and *X-RED32*; Stoe & Cie, 2008 ▶) *T*_min_ = 0.493, *T*_max_ = 0.748
10827 measured reflections
2998 independent reflections
2556 reflections with *I* > 2σ(*I*)
*R*_int_ = 0.032

#### Refinement

*R*[*F* ^2^ > 2σ(*F* ^2^)] = 0.042
*wR*(*F* ^2^) = 0.094
*S* = 1.06
2998 reflections
179 parameters
H-atom parameters constrained
Δρ_max_ = 0.56 e Å^−3^
Δρ_min_ = −0.58 e Å^−3^

### 

Data collection: *X-AREA* (Stoe & Cie, 2008 ▶); cell refinement: *X-AREA*; data reduction: *X-AREA*; program(s) used to solve structure: *SHELXS97* (Sheldrick, 2008 ▶); program(s) used to refine structure: *SHELXL97* (Sheldrick, 2008 ▶); molecular graphics: *XP* in *SHELXTL* (Sheldrick, 2008 ▶) and *DIAMOND* (Brandenburg, 2011 ▶); software used to prepare material for publication: *XCIF* in *SHELXTL*.

## Supplementary Material

Crystal structure: contains datablock(s) I, global. DOI: 10.1107/S1600536813007150/bt6898sup1.cif

Structure factors: contains datablock(s) I. DOI: 10.1107/S1600536813007150/bt6898Isup2.hkl

Additional supplementary materials:  crystallographic information; 3D view; checkCIF report

## Figures and Tables

**Table 1:** Selected bond lengths (Å)

Mn1—N1	2.180 (3)
Mn1—O1	2.211 (2)
Mn1—N10	2.322 (2)

**Table 2:** Hydrogen-bond geometry (Å, °)

*D*—H⋯*A*	*D*—H	H⋯*A*	*D*⋯*A*	*D*—H⋯*A*
O1—H1*O*1⋯N20	0.82	1.92	2.731 (3)	173

**Fig. 1.**
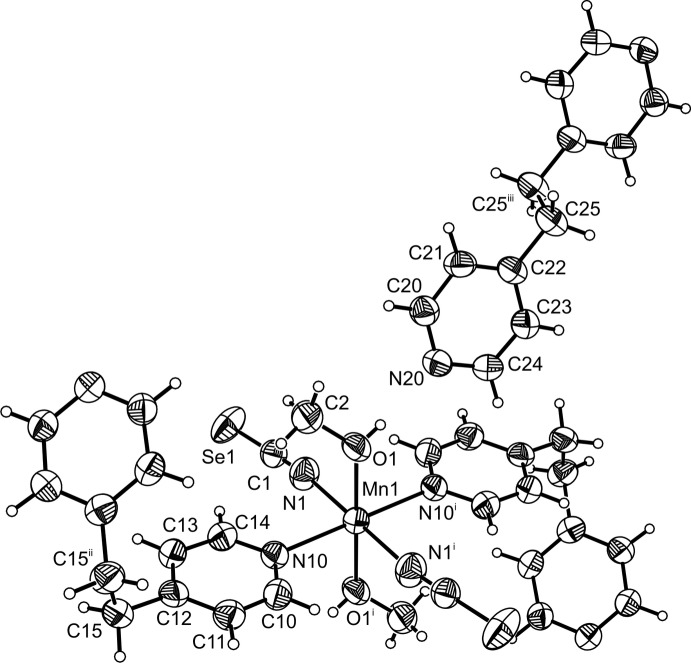
The crystal structure of the title compound with labeling and displacement ellipsoids drawn at the 50% probability level. [Symmetry codes: (i) -x,+1, -y+2, -z+1; (ii) -x+1, y, -z + 3/2; (iii) -x+1/2, -y+1/2, -z+1.]

**Fig. 2.**
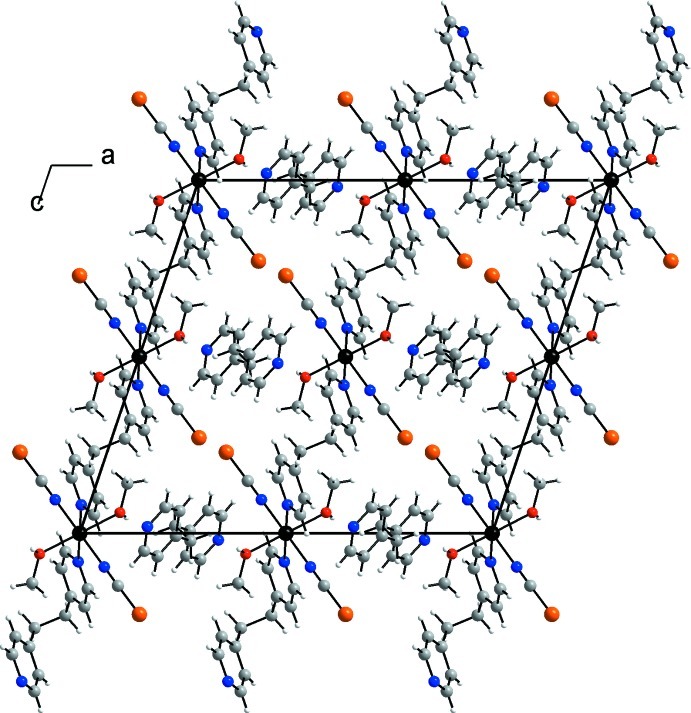
The crystal structure of the title compound with view along the b-axis (black = manganese, blue = nitrogen, orange = selenium, red = oxygen, grey = carbon, white = hydrogen).

**Fig. 3.**
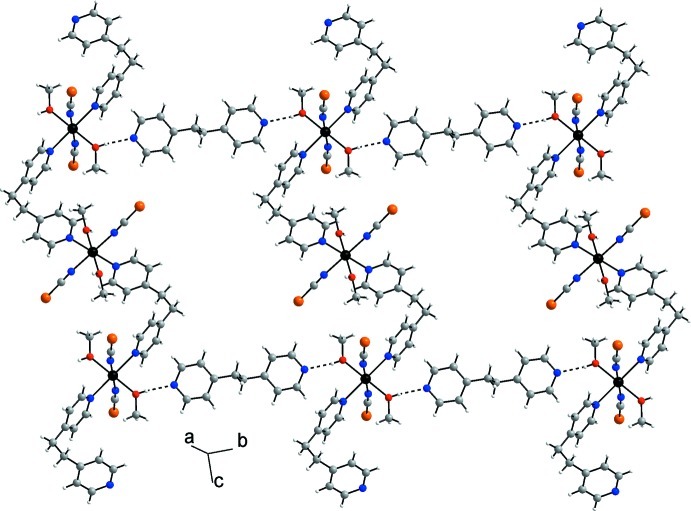
The crystal structure of the title compound with O—H···N hydrogen bonds shown as dashed lines (black = manganese, blue = nitrogen, orange = selenium, red = oxygen, grey = carbon, white = hydrogen).

## supplementary crystallographic information

### Comment

Recently, we have reported on the synthesis and characterization of thiocyanate coordination polymers with monodentate and bidentate neutral co-ligands like *e.g.* pyridine, pyridazine or 1,2-bis(pyridin-4-yl)ethylene (Boeckmann & Näther, 2010, 2012; Wöhlert & Näther, 2012*a*; Wöhlert & Näther, 2012*b*). Within this project we investigated the influence of the neutral co-ligand on the structural, thermal and magnetic properties of such compounds. In further work we also investigated the influence of the anionic ligand. In this context we have reported a new coordination polymer based on cobalt(II) selenocyanate and 1,2-bis(pyridin-4-yl)ethylene, in which the cobalt(II) cations are connected by the selenocyanato anions into chains that are further linked into layers by the neutral N-donor co-ligand (Wöhlert *et al.*, 2012). In the present investigation we tried to prepare similar compounds with manganese(II) selenocyanate and 1,2-bis(pyridin-4-yl)ethane (bpa), which results in the formation of single-crystals of the title compound.

The asymmetric unit of the title compound [Mn(NCSe)_2_(C_12_H_12_N_2_)(CH_3_OH)_2_]*_n_*^.^*n*C_12_H_12_N_2_ solvate consists of a manganese(II) cation and one non-coordinating bpa ligand which are located on a center of inversion, one coordinating bpa ligand on a 2-fold rotation axis and one selenocyanate anion and one methanol molecule in general positions (Fig. 1). In the crystal structure each manganese(II) cation is coordinated by two terminal *N*-bonded selenocyanate anions, two *O*-bonded methanol molecules and two *N*-bonded bpa ligands within slightly distorted octahedra. The MnN_4_O_2_ distances ranges from 2.180 (3) Å to 2.322 (2) Å with angles around the manganese(II) cation between 88.87 (9) ° to 91.13 (9) ° and of 180 ° (Tab. 1). The manganese(II) cations are linked by the bpa ligands into chains which elongate in the direction of the crystallographic *c*-axis (Fig. 2). These chains are further linked into layers parallel to the (3 -1 0) plane by non-coordinated bpa molecules *via* O–H–N hydrogen bonding (Fig. 3).

### Experimental

MnCl_2_x2H_2_O, KNCSe and 1,2-bis(pyridin-4-yl)ethane were obtained from Alfa Aesar. All chemicals were used without further purification. 0.15 mmol (24 mg) MnCl_2_x2H_2_O and 0.2 mmol (28 mg) KNCSe were reacted with 0.6 mmol (109 mg) 1,2-bis(pyridin-4-yl)ethane in 1 ml methanol. Light-red single crystals of the title compound were obtained after one week.

### Refinement

The C—H H atoms were positions with idealized geometry (methyl H atoms allowed to rotate but not to tip) and were refined with *U*_iso_(H) = 1.2 *U*_eq_(C) (1.5 for methyl H atoms) using a riding model with C—H = 0.93 Å, C—H_2_ = 0.97 Å and C—H_3_ = 0.96 Å. The O—H H atom of the methanol molecule was located in difference map, its bond length was set to 0.82 Å and finally it was refined with *U*_iso_(H) = 1.2 *U*_eq_(O) using a riding model.

### Crystal data

**Table:**

[Mn(NCSe)_2_(C_12_H_12_N_2_)(CH_4_O)_2_]·C_12_H_12_N_2_	*F*(000) = 1404
*M**_r_* = 697.46	*D*_x_ = 1.501 Mg m^−^^3^
Monoclinic, *C*2/*c*	Mo *K*α radiation, λ = 0.71073 Å
Hall symbol: -C 2yc	Cell parameters from 10827 reflections
*a* = 19.184 (1) Å	θ = 2.2–26.0°
*b* = 9.7854 (4) Å	µ = 2.82 mm^−^^1^
*c* = 17.3468 (9) Å	*T* = 293 K
β = 108.624 (4)°	Block, light-red
*V* = 3085.9 (3) Å^3^	0.14 × 0.11 × 0.06 mm
*Z* = 4	

### Data collection

**Table:**

Stoe IPDS-2 diffractometer	2998 independent reflections
Radiation source: fine-focus sealed tube	2556 reflections with *I* > 2σ(*I*)
Graphite monochromator	*R*_int_ = 0.032
ω scan	θ_max_ = 26.0°, θ_min_ = 2.2°
Absorption correction: numerical (*X-SHAPE* and *X-RED32*; Stoe & Cie, 2008)	*h* = −23→23
*T*_min_ = 0.493, *T*_max_ = 0.748	*k* = −12→10
10827 measured reflections	*l* = −21→21

### Refinement

**Table:**

Refinement on *F*^2^	Primary atom site location: structure-invariant direct methods
Least-squares matrix: full	Secondary atom site location: difference Fourier map
*R*[*F*^2^ > 2σ(*F*^2^)] = 0.042	Hydrogen site location: inferred from neighbouring sites
*wR*(*F*^2^) = 0.094	H-atom parameters constrained
*S* = 1.06	*w* = 1/[σ^2^(*F*_o_^2^) + (0.0346*P*)^2^ + 5.6439*P*] where *P* = (*F*_o_^2^ + 2*F*_c_^2^)/3
2998 reflections	(Δ/σ)_max_ = 0.001
179 parameters	Δρ_max_ = 0.56 e Å^−^^3^
0 restraints	Δρ_min_ = −0.58 e Å^−^^3^

### Special details

**Table:**

Geometry. All e.s.d.'s (except the e.s.d. in the dihedral angle between two l.s. planes) are estimated using the full covariance matrix. The cell e.s.d.'s are taken into account individually in the estimation of e.s.d.'s in distances, angles and torsion angles; correlations between e.s.d.'s in cell parameters are only used when they are defined by crystal symmetry. An approximate (isotropic) treatment of cell e.s.d.'s is used for estimating e.s.d.'s involving l.s. planes.
Refinement. Refinement of *F*^2^ against ALL reflections. The weighted *R*-factor *wR* and goodness of fit *S* are based on *F*^2^, conventional *R*-factors *R* are based on *F*, with *F* set to zero for negative *F*^2^. The threshold expression of *F*^2^ > σ(*F*^2^) is used only for calculating *R*-factors(gt) *etc*. and is not relevant to the choice of reflections for refinement. *R*-factors based on *F*^2^ are statistically about twice as large as those based on *F*, and *R*- factors based on ALL data will be even larger.

### Fractional atomic coordinates and isotropic or equivalent isotropic displacement parameters (Å^2^)

**Table:**

	*x*	*y*	*z*	*U*_iso_*/*U*_eq_	
Mn1	0.5000	1.0000	0.5000	0.04285 (17)	
N1	0.58695 (16)	0.8976 (3)	0.59516 (18)	0.0613 (7)	
C1	0.63537 (18)	0.8574 (3)	0.64832 (19)	0.0490 (7)	
Se1	0.71095 (2)	0.79679 (6)	0.72941 (2)	0.07953 (18)	
N10	0.51948 (13)	1.1948 (3)	0.58050 (15)	0.0456 (6)	
C10	0.48071 (18)	1.3084 (3)	0.55279 (19)	0.0519 (7)	
H10	0.4496	1.3092	0.4992	0.062*	
C11	0.48422 (19)	1.4238 (3)	0.59895 (19)	0.0538 (7)	
H11	0.4558	1.4997	0.5765	0.065*	
C12	0.52999 (17)	1.4274 (3)	0.67895 (18)	0.0479 (7)	
C13	0.57134 (18)	1.3113 (3)	0.70726 (19)	0.0506 (7)	
H13	0.6036	1.3087	0.7602	0.061*	
C14	0.56489 (18)	1.1998 (3)	0.65745 (18)	0.0503 (7)	
H14	0.5936	1.1235	0.6782	0.060*	
C15	0.5318 (2)	1.5500 (3)	0.7320 (2)	0.0579 (8)	
H15B	0.5292	1.6324	0.7002	0.070*	
H15A	0.5782	1.5512	0.7761	0.070*	
N20	0.34138 (14)	0.6923 (3)	0.51881 (16)	0.0499 (6)	
C20	0.31374 (17)	0.6393 (3)	0.57385 (19)	0.0504 (7)	
H20	0.3201	0.6876	0.6218	0.060*	
C21	0.27652 (18)	0.5175 (3)	0.5634 (2)	0.0525 (8)	
H21	0.2592	0.4845	0.6042	0.063*	
C22	0.26479 (16)	0.4436 (3)	0.49260 (19)	0.0484 (7)	
C23	0.29261 (19)	0.4991 (4)	0.4349 (2)	0.0556 (8)	
H23	0.2864	0.4537	0.3861	0.067*	
C24	0.32931 (18)	0.6210 (4)	0.4504 (2)	0.0556 (8)	
H24	0.3470	0.6565	0.4105	0.067*	
C25	0.22544 (19)	0.3082 (3)	0.4790 (2)	0.0594 (8)	
H25B	0.2067	0.2898	0.4211	0.071*	
H25A	0.1838	0.3135	0.4991	0.071*	
O1	0.41951 (12)	0.9297 (2)	0.55852 (13)	0.0569 (6)	
H1O1	0.3981	0.8571	0.5437	0.085*	
C2	0.4253 (2)	0.9473 (4)	0.6411 (2)	0.0733 (11)	
H2A	0.4674	0.8985	0.6747	0.110*	
H2B	0.3817	0.9126	0.6502	0.110*	
H2C	0.4305	1.0427	0.6546	0.110*	

### Atomic displacement parameters (Å^2^)

**Table:**

	*U*^11^	*U*^22^	*U*^33^	*U*^12^	*U*^13^	*U*^23^
Mn1	0.0432 (3)	0.0442 (3)	0.0405 (3)	−0.0021 (3)	0.0125 (3)	−0.0006 (3)
N1	0.0589 (17)	0.0628 (18)	0.0552 (16)	0.0070 (14)	0.0083 (14)	0.0008 (14)
C1	0.0547 (18)	0.0482 (17)	0.0464 (17)	−0.0017 (14)	0.0194 (15)	−0.0048 (13)
Se1	0.0631 (2)	0.1235 (4)	0.0473 (2)	0.0165 (2)	0.01114 (17)	0.0192 (2)
N10	0.0458 (13)	0.0472 (14)	0.0446 (13)	−0.0059 (11)	0.0153 (11)	−0.0015 (11)
C10	0.0552 (18)	0.0522 (18)	0.0444 (16)	0.0001 (15)	0.0105 (14)	−0.0015 (14)
C11	0.0618 (19)	0.0460 (18)	0.0528 (18)	0.0041 (15)	0.0171 (15)	0.0027 (14)
C12	0.0551 (17)	0.0460 (17)	0.0474 (16)	−0.0100 (14)	0.0231 (14)	−0.0007 (13)
C13	0.0529 (17)	0.0551 (19)	0.0411 (15)	−0.0065 (15)	0.0113 (13)	0.0002 (14)
C14	0.0531 (17)	0.0491 (17)	0.0471 (17)	0.0008 (14)	0.0139 (14)	0.0028 (14)
C15	0.078 (2)	0.0452 (17)	0.0534 (18)	−0.0115 (16)	0.0252 (17)	−0.0031 (15)
N20	0.0517 (14)	0.0441 (14)	0.0524 (15)	−0.0061 (12)	0.0144 (12)	0.0033 (11)
C20	0.0539 (18)	0.0471 (17)	0.0509 (17)	−0.0018 (14)	0.0179 (15)	−0.0029 (14)
C21	0.0586 (18)	0.0501 (19)	0.0542 (18)	−0.0050 (15)	0.0258 (15)	0.0060 (14)
C22	0.0445 (16)	0.0423 (15)	0.0574 (18)	−0.0046 (13)	0.0150 (14)	0.0021 (14)
C23	0.064 (2)	0.0557 (18)	0.0476 (17)	−0.0113 (17)	0.0184 (15)	−0.0044 (15)
C24	0.0605 (19)	0.058 (2)	0.0504 (17)	−0.0138 (16)	0.0208 (15)	0.0050 (15)
C25	0.0547 (19)	0.0495 (19)	0.073 (2)	−0.0122 (15)	0.0186 (17)	−0.0020 (16)
O1	0.0645 (14)	0.0578 (13)	0.0537 (12)	−0.0239 (11)	0.0262 (11)	−0.0092 (11)
C2	0.088 (3)	0.083 (3)	0.056 (2)	−0.030 (2)	0.032 (2)	−0.0117 (19)

### Geometric parameters (Å, º)

**Table:**

Mn1—N1^i^	2.180 (3)	C15—H15A	0.9700
Mn1—N1	2.180 (3)	N20—C24	1.331 (4)
Mn1—O1^i^	2.211 (2)	N20—C20	1.336 (4)
Mn1—O1	2.211 (2)	C20—C21	1.372 (4)
Mn1—N10^i^	2.322 (2)	C20—H20	0.9300
Mn1—N10	2.322 (2)	C21—C22	1.380 (5)
N1—C1	1.149 (4)	C21—H21	0.9300
C1—Se1	1.769 (3)	C22—C23	1.386 (4)
N10—C10	1.338 (4)	C22—C25	1.506 (4)
N10—C14	1.342 (4)	C23—C24	1.368 (5)
C10—C11	1.374 (4)	C23—H23	0.9300
C10—H10	0.9300	C24—H24	0.9300
C11—C12	1.386 (4)	C25—C25^iii^	1.509 (7)
C11—H11	0.9300	C25—H25B	0.9700
C12—C13	1.383 (5)	C25—H25A	0.9700
C12—C15	1.506 (4)	O1—C2	1.412 (4)
C13—C14	1.372 (4)	O1—H1O1	0.8200
C13—H13	0.9300	C2—H2A	0.9600
C14—H14	0.9300	C2—H2B	0.9600
C15—C15^ii^	1.538 (7)	C2—H2C	0.9600
C15—H15B	0.9700		
			
N1^i^—Mn1—N1	180.000 (1)	C12—C15—H15B	109.1
N1^i^—Mn1—O1^i^	89.25 (10)	C15^ii^—C15—H15B	109.1
N1—Mn1—O1^i^	90.75 (10)	C12—C15—H15A	109.1
N1^i^—Mn1—O1	90.75 (10)	C15^ii^—C15—H15A	109.1
N1—Mn1—O1	89.25 (10)	H15B—C15—H15A	107.9
O1^i^—Mn1—O1	180.0	C24—N20—C20	116.0 (3)
N1^i^—Mn1—N10^i^	89.13 (10)	N20—C20—C21	123.5 (3)
N1—Mn1—N10^i^	90.87 (10)	N20—C20—H20	118.3
O1^i^—Mn1—N10^i^	88.92 (8)	C21—C20—H20	118.3
O1—Mn1—N10^i^	91.08 (8)	C20—C21—C22	120.2 (3)
N1^i^—Mn1—N10	90.87 (10)	C20—C21—H21	119.9
N1—Mn1—N10	89.13 (10)	C22—C21—H21	119.9
O1^i^—Mn1—N10	91.08 (8)	C21—C22—C23	116.5 (3)
O1—Mn1—N10	88.92 (8)	C21—C22—C25	122.1 (3)
N10^i^—Mn1—N10	180.0	C23—C22—C25	121.4 (3)
C1—N1—Mn1	172.7 (3)	C24—C23—C22	119.5 (3)
N1—C1—Se1	179.0 (3)	C24—C23—H23	120.2
C10—N10—C14	115.9 (3)	C22—C23—H23	120.2
C10—N10—Mn1	120.1 (2)	N20—C24—C23	124.3 (3)
C14—N10—Mn1	123.9 (2)	N20—C24—H24	117.8
N10—C10—C11	123.7 (3)	C23—C24—H24	117.8
N10—C10—H10	118.1	C22—C25—C25^iii^	112.6 (3)
C11—C10—H10	118.1	C22—C25—H25B	109.1
C10—C11—C12	120.1 (3)	C25^iii^—C25—H25B	109.1
C10—C11—H11	119.9	C22—C25—H25A	109.1
C12—C11—H11	119.9	C25^iii^—C25—H25A	109.1
C13—C12—C11	116.3 (3)	H25B—C25—H25A	107.8
C13—C12—C15	122.6 (3)	C2—O1—Mn1	126.0 (2)
C11—C12—C15	121.1 (3)	C2—O1—H1O1	107.0
C14—C13—C12	120.2 (3)	Mn1—O1—H1O1	118.7
C14—C13—H13	119.9	O1—C2—H2A	109.5
C12—C13—H13	119.9	O1—C2—H2B	109.5
N10—C14—C13	123.7 (3)	H2A—C2—H2B	109.5
N10—C14—H14	118.1	O1—C2—H2C	109.5
C13—C14—H14	118.1	H2A—C2—H2C	109.5
C12—C15—C15^ii^	112.4 (2)	H2B—C2—H2C	109.5

Symmetry codes: (i) −*x*+1, −*y*+2, −*z*+1; (ii) −*x*+1, *y*, −*z*+3/2; (iii) −*x*+1/2, −*y*+1/2, −*z*+1.

### Hydrogen-bond geometry (Å, º)

**Table:**

*D*—H···*A*	*D*—H	H···*A*	*D*···*A*	*D*—H···*A*
O1—H1*O*1···N20	0.82	1.92	2.731 (3)	173
